# The use of outdoor environments in school-based physical education: a scoping review

**DOI:** 10.3389/fspor.2026.1810598

**Published:** 2026-06-23

**Authors:** Øyvind Bjerke, Thomas Priqueler

**Affiliations:** 1 Department of Teacher Education, Norwegian University of Science and Technology, Trondheim, Norway; 2 Department of Sport Science and Physical Education, Ecole Normale Superieure de Rennes, Bruz, France

**Keywords:** children and youth, friluftsliv, learning environment, outdoor education, pedagogy

## Abstract

**Background:**

Physical education (PE) is a compulsory school subject with responsibility for promoting physical activity, learning, and broader educational aims, including wellbeing and sustainability. Outdoor environments are increasingly recognized as pedagogically valuable settings for PE, but research on outdoor PE is dispersed across multiple traditions and lacks a consolidated overview.

**Objective:**

This scoping review aimed to map the extent, nature, and characteristics of empirical research examining the use of outdoor environments in compulsory school-based PE.

**Methods:**

The review followed Joanna Briggs Institute (JBI) methodological guidance and was reported in accordance with PRISMA-ScR. Peer-reviewed empirical studies published in Scopus or EBSCO from 2005 onwards in English, French, or Norwegian were eligible if they investigated outdoor environments as a central component of school-based PE.

**Results:**

Forty-one studies published between 2005 and 2025 were included. Research was geographically concentrated in Western Europe and the United States and primarily focused on primary and lower secondary pupils. Four outcome domains were identified: physical; psychological/emotional; social/behavioral; and environmental. Pedagogical purposes ranged from promoting physical activity and fitness to supporting experimental learning, social development, safety competence, and environmental education. Activities included fitness and games, orienteering and navigation-based tasks, cycling, adventure challenges, plogging, and blue-space activities. Outdoor environments comprised school grounds, parks and forests, beaches, and blue-space settings.

**Conclusions:**

The literature on outdoor PE has expanded but remains uneven in scope and focus, privileging physical outcomes over pedagogical processes and sustainability-related learning. Future research should enhance conceptual clarity, address under-represented age groups and contexts, and more explicitly examine pedagogical mechanisms alongside health, learning, and environmental outcomes.

## Introduction

Physical Education (PE) is a compulsory subject in school and plays a unique role in promoting lifelong participation in physical activity through physical, social, cognitive, and affective development (1). Over recent decades, the subject has moved away from its original aims. PE was initially introduced in schools with the aim of preparing young people for military service (2). Contemporary curricula, however, emphasize lifelong participation in physical activity, alongside physical, social, cognitive, and affective development (3). International policy documents from OECD (1) and UNESCO (4) highlight the importance of providing meaningful movement opportunities that support learners' experiences across a range of learning environments, where outdoor environments in PE have emerged as a promising pedagogical domain for achieving broader educational, health-related, and sustainability-oriented aims.

Despite benefits of the outdoors, school-based PE continues to be delivered predominantly in indoor settings. Studies from Norway (5) and other European contexts indicate a longstanding tendency to situate the PE in gymnasiums, sports halls, and other structured indoor environments. This practice has been linked to traditions, curricular interpretations, access to facilities, and perceptions of classroom management and control (6). However, policy documents provide teachers with substantial autonomy in *where* the education should take place (7). Hence, great variations in location of PE exists between schools, countries and nations regarding the extent to which outdoor areas that is used, such as school yards, local parks, beaches, forests and municipal sport facilities.

There is growing scientific interest in how natural, semi-natural, and built outdoor environments influence children's physical activity behavior (8), learning processes (9) environmental attitudes (10), and psychological wellbeing (11). Research conducted outside the PE field, such as “green exercise” (12), nature-based learning (13), and outdoor education (14), suggest that physical activity in outdoor settings are associated with higher levels of moderate-to-vigorous physical activity (MVPA), including enhanced affective and cognitive functioning, reduced stress, and stronger connections to nature (11, 13, 15, 16).

Although numerous empirical studies have examined specific outdoor practices in PE, such as orienteering (17), nature-based activities (18), cycling programs (19), plogging (jogging and litter collection) (20), or water-based learning (21), the overall body of research remains fragmented. Studies differ substantially in their theoretical orientations, methodological approaches, activity types, outcome measures. Moreover, there are considerably variations in how *outdoor PE* is conceptualized, reflecting overlap with adjacent educational and physical activity traditions. For example, *outdoor education* is often associated with the Scandinavian concept of *friluftsliv*, which in some national curricula (e.g., Norway) is formally embedded within the PE subject. In contrast, *uteskole* (“outdoor school”) refers to a broader pedagogical model that involves teaching multiple school subjects outdoors and is *not* specific to PE (22). Other strands of the literature draw on related but distinct concepts, including *nature-based physical activity* and *outdoor adventure education*, which typically emphasize experiential learning, challenge, and personal or social development rather than PE-specific curricular aims (23). Together, these overlapping, but conceptually distinct traditions, contribute to ambiguity regarding what constitutes outdoor PE as a pedagogical practice.

Despite an increasing number of empirical studies across these related fields, no comprehensive review has systematically mapped the extent, nature, and characteristics of empirical research on outdoor *physical education*. Existing reviews have focused on adjacent topics such as green exercise (12, 16), outdoor education in general (14), school-based physical activity promotion (24), or adventure education (25). To date, there is no synthesis that addresses outdoor environments *specifically within school-based PE lessons*, nor one that systematically examines questions such as how environments are used pedagogically, what variables are studied, what age groups are targeted, which activity types dominate, and in what kinds of settings interventions take place. This conceptual fragmentation is not merely an academic inconvenience; it directly impedes the development of evidence-based curricula and coherent pedagogical guidance for teachers seeking to integrate outdoor environments into their practice.

Addressing these questions is essential for several reasons. First, synthesizing this literature can clarify how outdoor environments may contribute to PE's core curricular aims, such as promoting physical activity, developing fundamental movement skills, supporting social learning, and fostering positive attitudes toward the environment. Second, identifying patterns and gaps in existing research may support teachers and curriculum developers seeking to integrate outdoor environments more systematically into PE practice. Third, as sustainability and environmental citizenship gain prominence in educational policy, PE is increasingly positioned as a subject that can cultivate ecological awareness and responsibility. Understanding how outdoor spaces are currently used to support such aims is therefore timely. Finally, a comprehensive mapping of the research can illuminate gaps in evidence, such as under-represented age groups, under-studied activity types, or under-examined outcome domains, thereby guiding future research agendas.

Given the breadth, heterogeneity, and conceptual diversity of the existing literature, a scoping review is the most appropriate methodological approach. Scoping reviews are particularly useful for mapping the key concepts underpinning a research area, identifying the types and sources of evidence available, and clarifying how research has been conducted in a field where definitions and practices vary widely (26). Following methodological guidance of the Joanna Briggs Institute (JBI) and reporting standards such as PRISMA-ScR (27, 28), this scoping review aims to provide a systematic overview of the research landscape without evaluating intervention effectiveness. Instead, the review seeks to enhance conceptual clarity and provide a foundation for future empirical and theoretical work on outdoor PE.

### Review questions

Based on the background and the evidence, the present scoping review aims to map and synthesize the extent, nature, and characteristics of empirical research on the use of outdoor environments in school-based PE. Specifically, the review seeks to identify:

1. Which outcome domains are examined?
2. What pedagogical purposes underpin outdoor PE interventions?
3. Which activity types and outdoor environments are represented?
4. Which educational levels and age groups are represented?
5. Where are the significant evidence gaps in the existing evidence base?

While the first four questions address the scope and nature of what has been studied, the fifth question spans all dimensions of the review; gaps may be evident not only across age groups but also in relation to geography, methodology, outcome domains, and activity types. Together, these questions are designed to map the breadth and diversity of the existing literature rather than evaluate its adequacy. In doing so, they also probe the extent to which outdoor PE research has moved beyond physical activity outcomes to address holistic educational aims, including psychological, social, and environmental dimensions of learning. By systematically mapping these dimensions, this scoping review provides a comprehensive overview of how outdoor environments have been incorporated into school-based PE over the past two decades. The findings contribute to a more coherent understanding of the field, highlight emerging themes and gaps, and offer insights that can support educators, curriculum developers, and researchers in leveraging the potential of outdoor PE to promote health, learning, and sustainability.

### Inclusion criteria

#### Participants

Given the focus on PE as a school subject, the participants included are children and adolescents enrolled in compulsory school-based PE programs, from primary school to upper secondary school (i.e., from early elementary levels to the end of high school). This includes all educational stages where PE is part of the school curriculum. Studies focusing on pre-school children (pre-primary education), university students, trainee teachers or teachers, not providing specific data on primary or secondary school pupils, were excluded.

#### Concept

The key concept is outdoor PE, understood as the intentional and pedagogically structured use of outdoor environments to deliver PE as part of the formal school curriculum. This includes, but is not limited to, other concepts such as green exercise, blue exercise, outdoor education, which is performed as a PE intervention. Studies were excluded if the outdoor environment was merely incidental or not central to the PE intervention. This includes studies focusing solely on unstructured outdoor play, recess, or general physical activity promotion without a clear pedagogical link to PE. Studies conducted in non-school settings, such as extracurricular programs, nature camps, or outdoor education initiatives not embedded in the PE curriculum, were also excluded.

#### Context

The study sought evidence presented in the context of school-based PE lessons delivered as part of the curriculum, in which the outdoors (e.g., natural, semi-natural, or built environments) is used as the setting. This includes schoolyards, sports fields, parks, forests, beaches, and other outdoor spaces used during PE classes. Studies conducted in non-school settings (e.g., after-school clubs, summer camps or community sports programs) and studies on the use of the outdoors not explicitly related to PE were excluded.

#### Sources of evidence

Sources of evidence published from January 1, 2005, reporting on empirical studies (including quantitative, qualitative, or mixed-methods studies), written in English, French and Norwegian, appearing in peer-reviewed journals and focusing on outdoor PE interventions as a core component of the study were considered. We did not include grey literature, non-empirical studies (including opinion pieces, editorials, and narrative reviews), studies that did not involve outdoor intervention in PE, and studies that were not peer-reviewed. A critical appraisal was not conducted as the purpose of scoping review is to map the extent and nature of available evidence rather than to assess methodological quality (26, 28).

## Methods

This review was guided by the JBI methodology for the conduct of scoping reviews and has been reported against PRISMA-ScR checklist (27, 28). A study protocol was created and registered in Open Science Framework (Registration https://doi.org/10.17605/OSF.IO/S8GKN).

### Search strategy

The search strategy aimed to locate peer-reviewed empirical studies. The search was carried out on 17 June 2025 using Scopus (for the Scopus database) and EBSCO (for the databases Education Source, ERIC, MEDLINE, Psychology and Behavioral Sciences Collection, and SPORTDiscus). Our search strategy was developed by identifying synonyms related to the concept of outdoor practice and including the term physical education as well as its acronym PE. The databases were chosen due to their relevance to the field of investigation, which is educational sciences, and to capture as many relevant articles as possible. The search strategy, including all identified keywords and index terms, was adapted for each included interface. The full search strings are provided in the appendix (Supplementary file 1), per PRISMA-ScR Item 8. The reference list of all included sources of evidence was screened for additional studies.

### Source screening and selection

**Figure 1:**
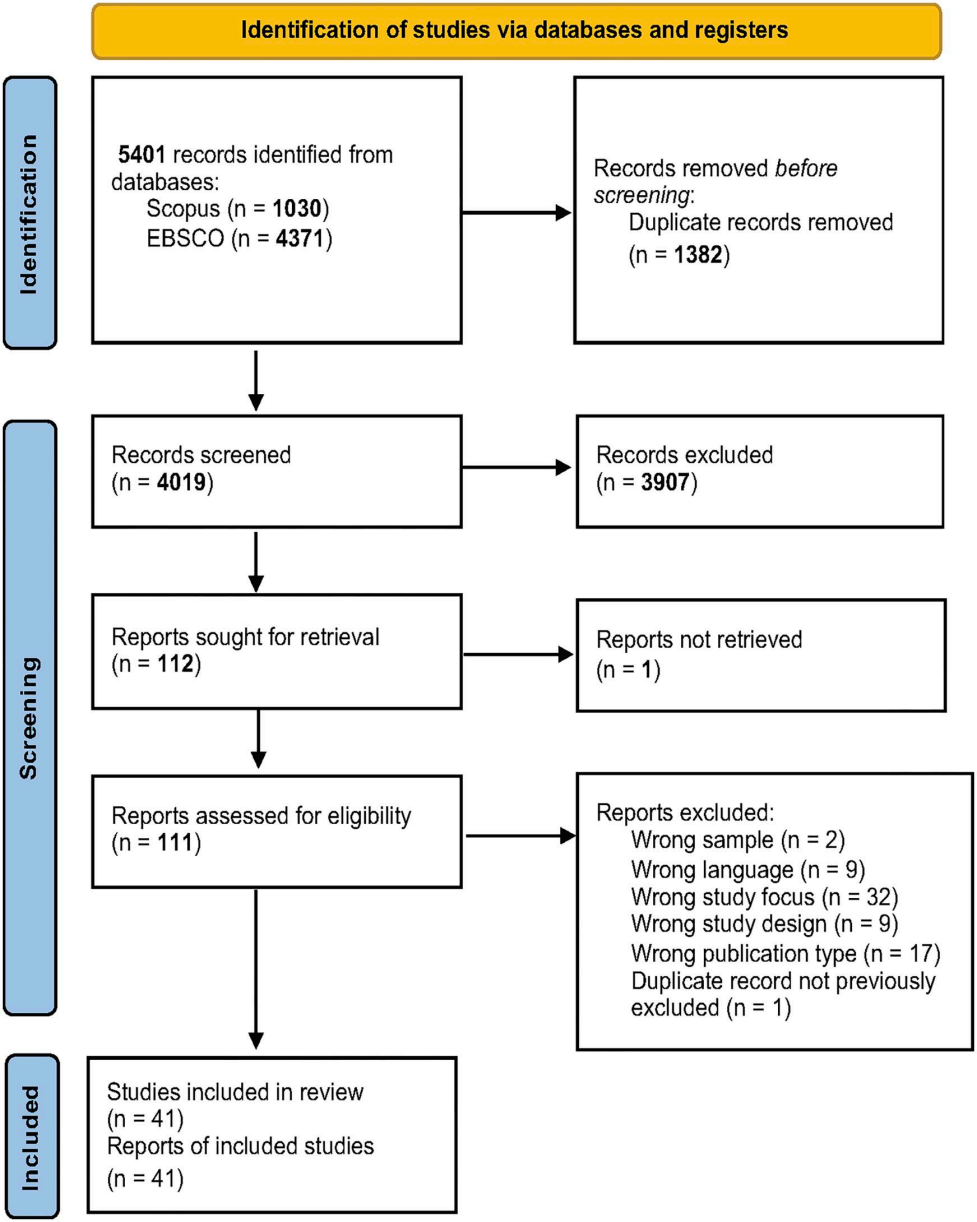
A PRISMA flow chart of the study selection and the screening processes [*From*: page et al. (56)].

Following the search, all 5,401 citations were collated and uploaded into the Deduplicator (https://tera-tools.com/deduplicator), and duplicates were removed. Screening of titles and abstracts was conducted by two reviewers with the aid of ASReview software (https://asreview.nl/) and following the ASReview recommendations. The screening procedure followed the protocol recommended by Quan et al. (29) and proceeded in three steps. First, a training set was established: five relevant studies were provided as prior knowledge to initialize the algorithm, five additional relevant studies were withheld as validation records to monitor retrieval performance, and ten irrelevant studies were used to train the classifier to distinguish relevant from irrelevant records. Second, the algorithm ranked all remaining records by predicted relevance, and both reviewers independently assessed these records in priority order, classifying each as relevant or irrelevant. Third, screening was discontinued once 100 consecutive irrelevant records had been identified, in accordance with a data-driven stopping rule (29). Full details of the procedure, including the training set composition and stopping rule rationale, are available in the registered study protocol on the Open Science Framework (https://doi.org/10.17605/OSF.IO/S8GKN). This screening phase was conducted independently by both reviewers, and any discrepancies in study inclusion were resolved through discussion. This semi-automated approach enhanced the efficiency of the screening process by prioritizing the most relevant records early in the review, while also minimizing the risk of human bias during the initial assessment of several thousand references (30). Full-text screening was then undertaken for all records deemed relevant at the previous stage. The full texts were assessed independently by both reviewers, and disagreements regarding inclusion were resolved through consensus. Figure 1 displays the study selection and the screening process in a PRISMA chart including the number of excluded and included studies at different stages of the process, and the reason for exclusion.

### Data extraction

Data relating to the review questions was extracted using a tailored data extraction template based on the JBI manual (28) and the recommendations of Pollock et al. (31). During the pilot extraction phase, two independent reviewers tested the data extraction form on the same five articles to ensure clarity and consistency. A single reviewer proceeded to complete extraction of all the other included studies. When uncertainty arose during data extraction, a second reviewer verified the extracted information checking the uncertain cases. The analysis was descriptive in terms of counts and categorization, any qualitative coding was used to support mapping (28).

## Results

This scoping review identified forty-one empirical studies published between January 1, 2005 and June 17, 2025, that examined the use of the outdoors in school-based PE. A summary of key study characteristics is presented in Supplementary Table S1 including citation information in Supplementary file 2.

### Geographical distribution and study design

**Figure 2:**
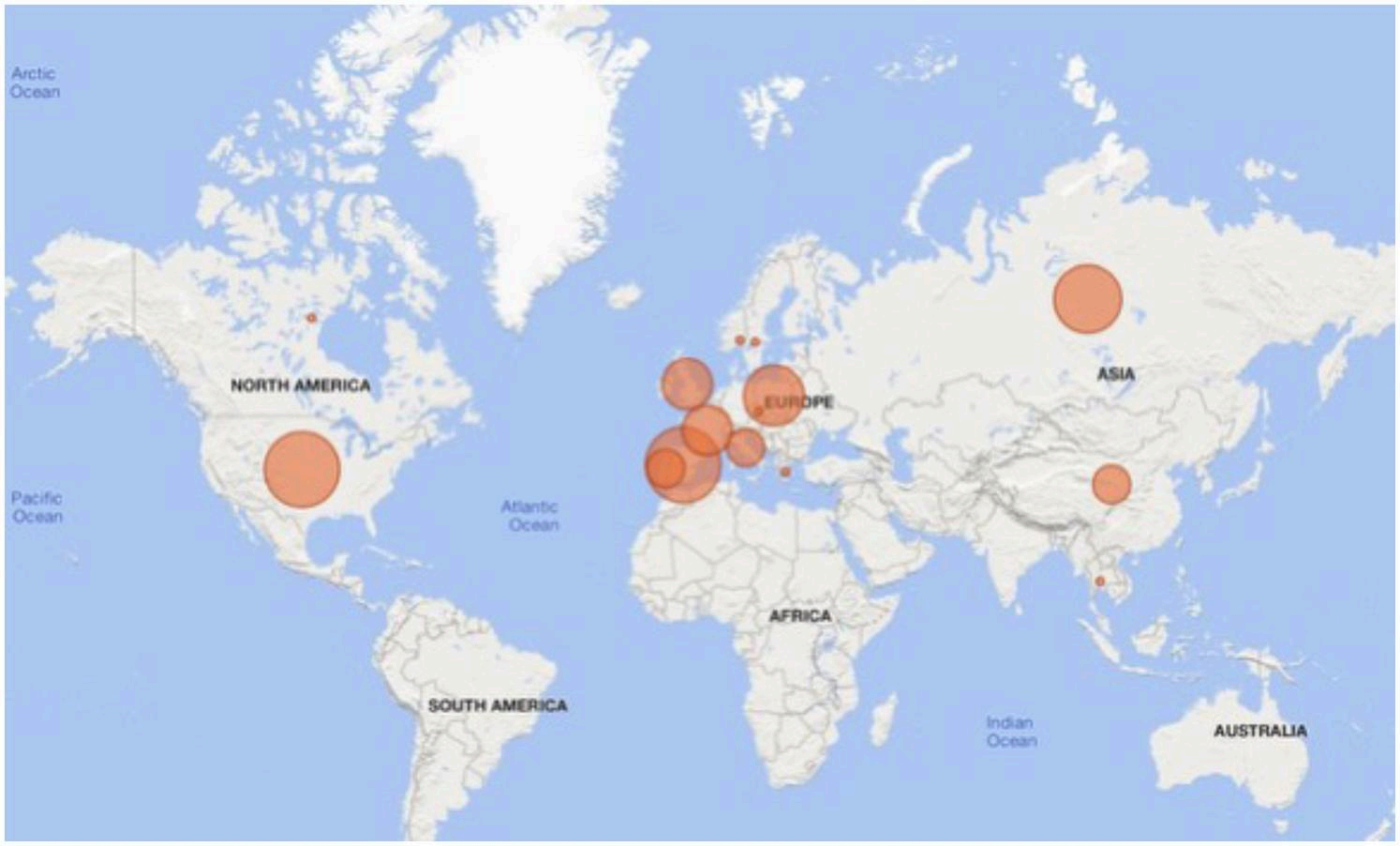
A heat map of the location of the included publications.

**Figure 3:**
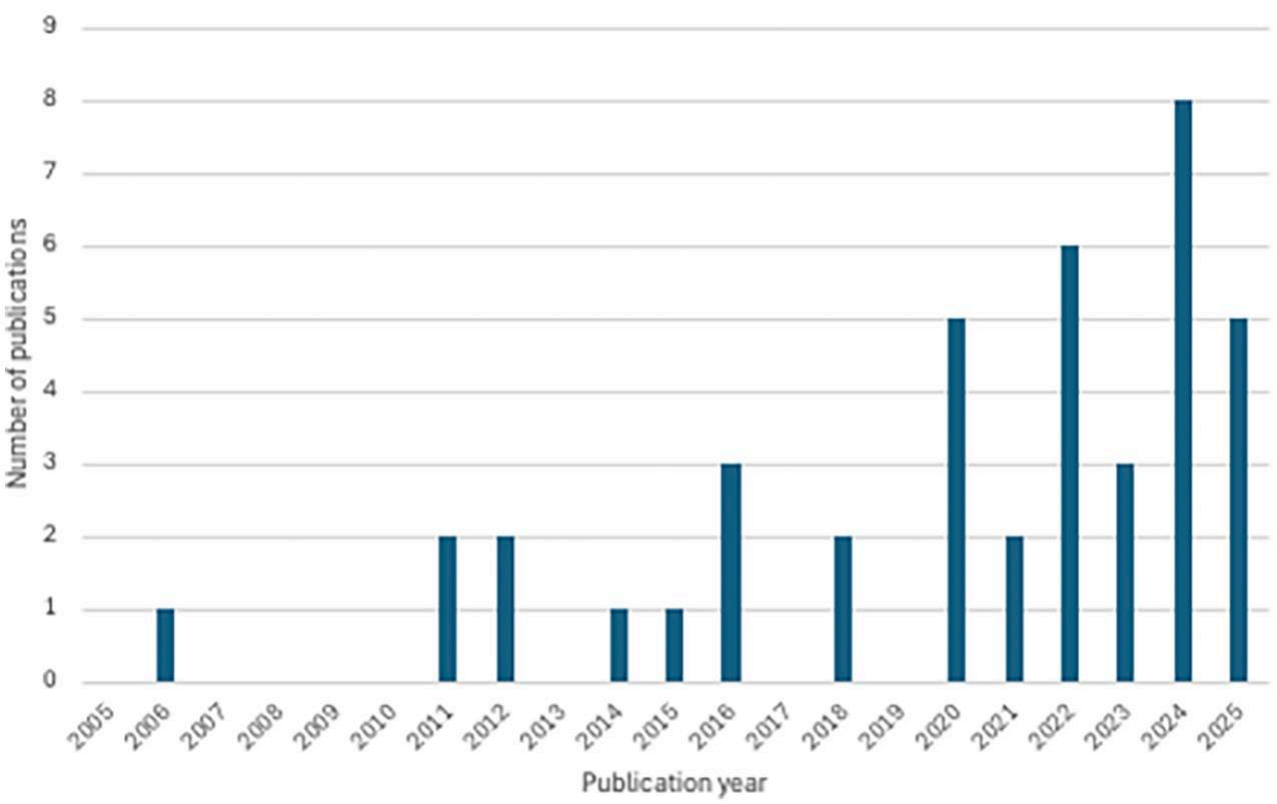
Number of studies by publication year.

The included studies were conducted across Europe, North America, and Asia, with a clear concentration in Western Europe and the United States. The geographical distribution of the included studies is illustrated as a heat map in Figure 2. Spain and the United States accounted for the highest number of studies, followed by Russia, Poland, France, and England. Only a small number of studies originated from Asia, and none were identified from Africa or South America. There were a higher number (29/41) of published papers from 2020 to 2025 as shown in Figure 3.

In terms of methodology, the evidence base was dominated by quantitative designs, particularly interventional and observational studies. These included randomized controlled trials, quasi-experimental studies, and large-scale observational studies using objective measures of physical activity (e.g., accelerometry or SOFIT). A smaller subset of studies employed qualitative or mixed-methods designs, primarily to explore students' experiences, social interactions, motivation, and learning processes in outdoor PE contexts.

Around half of the included studies (21/41) focused on primary and lower secondary school pupils, with children aged 7–12 years of age, being the most frequently studied group. Fewer studies targeted adolescents aged 13–15 years, and only a limited number included upper secondary school students (16–18 years). Overall, the literature shows a clear emphasis on younger age groups, with comparatively limited attention to older adolescents. It's noteworthy that most of the included studies are from Western Europe and from the USA. Supplementary Table S1 is a descriptive mapping tool, consistent with Peters et al. (28) summarizing the characteristics in the included papers.

### Outcome domains and variables examined

**Figure 4:**
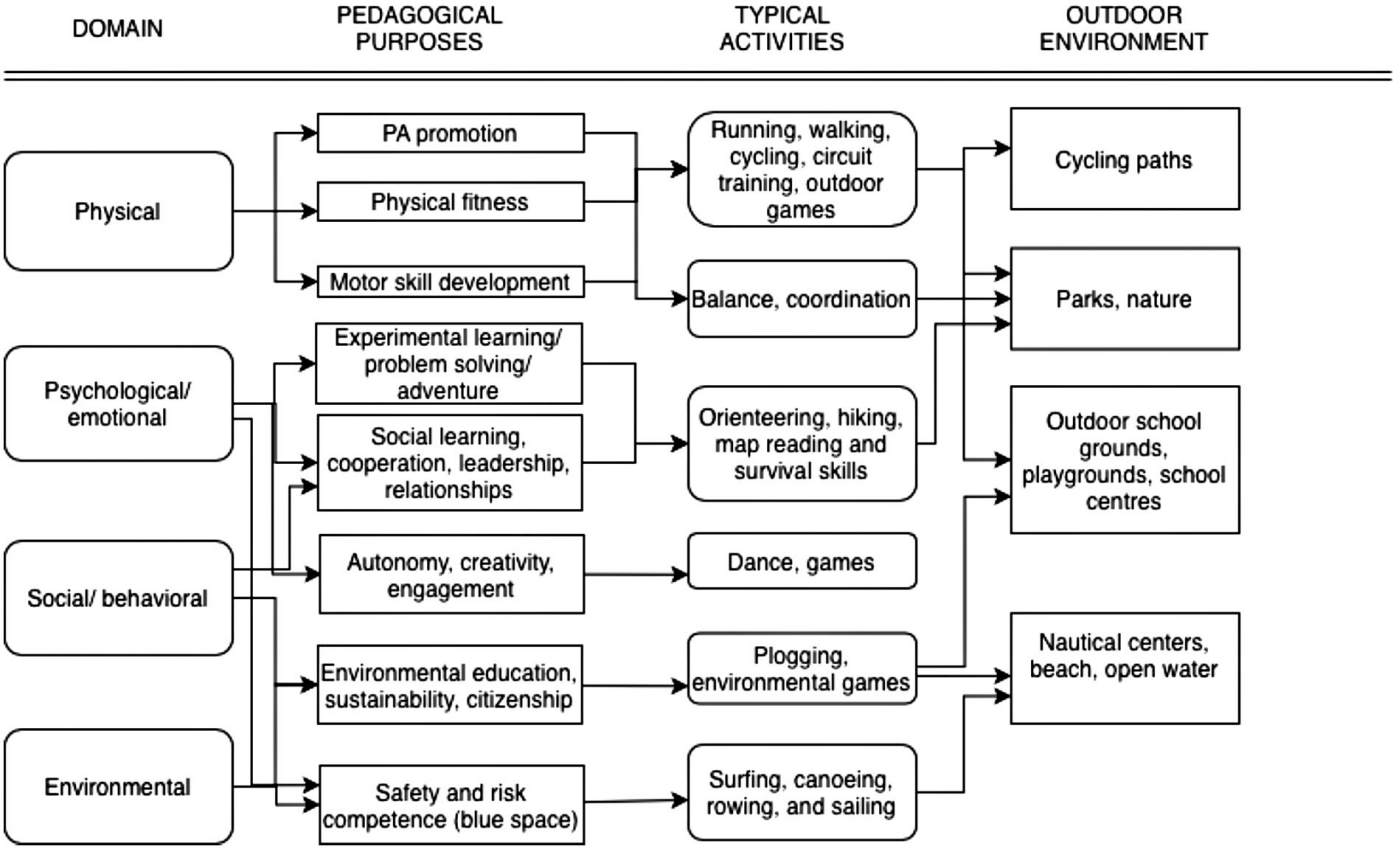
Mapping of outdoor PE characteristics across included studies.

Analysis of the included studies indicates that the outcome measures examined in relation to outdoor PE can be grouped into four overarching domains: physical, psychological and emotional, social and behavioral, and environmental. These domains reflect the range of constructs used to examine outcomes associated with outdoor PE. The mapping of the outcome domains including typical variables is shown in Figure 4.

The ***physical domain*** was most prominent and primarily focused on physical activity–related variables, particularly moderate-to-vigorous physical activity (MVPA), step counts, activity intensity, and sedentary time (32, 33). Many studies employed objective measurement tools such as accelerometry or systematic observation. In addition, several studies assessed components of physical fitness, including cardiorespiratory endurance, muscular strength, balance, agility, motor coordination, and anthropometric indicators such as body mass index, heart rate and body composition (34). A smaller number of studies reported physiological responses, including thermoregulation, hydration status, and fatigue-related measures (35).

The ***psychological and emotional domain*** included constructs related to students' motivational and affective experiences in outdoor PE contexts. Commonly examined variables were intrinsic motivation, enjoyment, self-efficacy, perceived autonomy support, perceived behavioral control, and intention to engage in physical activity (36, 37). Emotional outcomes such as stress, anxiety, confidence, body image, and body satisfaction were also reported, typically assessed through self-report instruments (38).

Outcomes within the ***social and behavioral domain*** were examined less frequently and were most often explored using qualitative or mixed-methods designs (39). Variables in this domain included peer cooperation, interaction dynamics, leadership, communication skills, sense of community, and disruptive behaviors, often in relation to pedagogical models emphasizing collaboration and group-based learning (37). The ***environmental domain*** focused on sustainability-related outcomes, including environmental awareness, environmental attitudes, ecological knowledge, and pro-environmental values (40). These variables were primarily examined in studies that explicitly integrated environmental content into PE lessons, such as plogging or environmentally themed outdoor activities (20).

Overall, the distribution of outcome domains demonstrates a strong emphasis on physical activity and fitness outcomes, alongside a more limited but emerging focus on psychological, social, and environmental dimensions of outdoor PE.

### Pedagogical purposes and activity types

The reviewed literature demonstrates that outdoor environments serve as a multifaceted educational setting and pedagogical purpose, often with overlapping purposes. A prominent purpose for using outdoor PE is to ***increase physical activity*** levels or to enhance physical fitness, often through activities as running, walking, cycling, circuit training or outdoor games compared to indoor alternatives (32, 41). Examples are also cycling interventions to promote for opportunities for daily physical activity engagement (33), while dance and games design are typically used for creative tasks (42).

Another pedagogical focus associated with outdoor PE is ***experimental learning and problem solving*** often through adventure and navigation-based activities (43). Particularly, orienteering is frequently implemented to enhance the pupil's skill within problem-solving, or to develop social skills (17). Other activities are hiking, map reading and/or survival skills. These activities were commonly linked to social learning outcomes, including cooperation, problem-solving, and motivation (43, 44).

A distinct group of studies examined blue-space activities, using activities such as surfing, canoeing, rowing, and sailing (39, 45). These interventions typically addressed combinations of physical, psychological, social, and ***safety-related outcomes***. In complementary programs focusing on outdoor swimming and water safety (SWS), these activities aim to equip learners with coping strategies of e.g., stress and safe behavior in natural, cold-water settings (21).

Finally, another purpose is activities with objectives that often extend beyond physical and psychological purposes to encompass broader civic and educational aims. One of these goals are ***environmental education and sustainability***, frequently using activities such as plogging or environmentally themed games to foster environmental awareness, deepen ecological knowledge, and civic responsibility (10).

### Types of outdoor environments

Outdoor PE interventions were conducted across a variety of settings. The outdoor environments include green and natural spaces, like parks and generalized natural environments used for fitness activities and orienteering (46). Another prominent use of the outdoor area is the built spaces and school-adjacent spaces, such as the outdoor school ground, playgrounds and the school centers (37). These areas include municipal sport centers, cycling paths and nearby urban outdoor spaces and facilities (47, 48). For activities in water and blue space areas are nautical centers, beaches and open water utilized for water-based activities (21, 39, 45).

## Discussion

The purpose of this scoping review was to map the extent, nature, and characteristics of empirical research examining the use of outdoor environments in school-based PE. By synthesizing evidence published between two decades, this scoping review provides an overview of how outdoor PE has been conceptualized, operationalized, and studied across different educational contexts. Rather than assessing effectiveness, the review sought to identify patterns, concentrations, and gaps in the existing literature.

### Scope and distribution of the literature

The findings indicate a growing body of research on outdoor PE, with a notable increase in publications over the past six years. This trend may reflect heightened awareness of the potential benefits associated with outdoor learning and activity (11). However, the research is unevenly distributed geographically and demographically. Most studies were conducted in Western Europe and North America, with relatively few originating from Asia and none from Africa or South America. This geographical concentration suggests that current knowledge about outdoor PE is shaped largely by specific curricular traditions, policy contexts, and cultural understandings of outdoor activity. Consequently, the transferability of findings to other educational systems and sociocultural contexts remains uncertain. The geographical concentration of this literature has implications that extend beyond sampling bias. The dominant frameworks in this literature, particularly the Scandinavian concept of *friluftsliv* (49), are deeply embedded in specific cultural relationships with nature, shaped by historical, climatic, and policy contexts that may not translate directly to other settings. Other regions encompass distinct but analogous traditions that link nature, physical activity, and education, including land-based learning in Indigenous communities (50, 51). For instance, in regions such as sub-Saharan Africa and South America, the limited presence of outdoor PE research may reflect the marginalization of locally grounded physical-cultural traditions within formal school curricula. The absence of comparative cross-cultural studies leaves fundamental questions unanswered: whether outcomes associated with outdoor PE in Western European settings generalize elsewhere, and what outdoor PE means pedagogically outside a Nordic or Anglo-American curricular context. Future research should therefore not only study outdoor PE in underrepresented regions but also engage with frameworks that are locally meaningful rather than imported. A more detailed country-level analysis of intervention scope and design, examining both where research originates and how outdoor PE is implemented within national curriculum contexts, would provide a useful direction for future work, particularly for those seeking to understand context-specific educational reform rather than overall trends.

The literature predominantly involves primary and lower secondary school pupils, involving few older adolescents, which aligns with similar scoping reviews (13). This difference may reflect curricular priorities, or historical constraints, or an assumption that the outdoor is more suitable for younger pupils. Nonetheless, it highlights an important gap in understanding how outdoor environments are used in PE across the full span of compulsory schooling.

Methodologically, the literature was dominated by quantitative and intervention-based designs, particularly studies examining physical activity levels and fitness-related outcomes. Qualitative and mixed-methods approaches were less frequently employed and were primarily used to explore students' experiences, social interactions, and motivational processes. This methodological pattern suggests that outdoor PE has most often been studied through a health- and activity-oriented lens, with comparatively less attention given to pedagogical processes and meaning-making.

### Outcome domains and pedagogical rationales

Across the included studies, physical activity and fitness outcomes were the most frequently examined variables. A consistent pattern across observational and interventional studies was the reporting of higher levels of physical activity, particularly MVPA, in outdoor settings compared to traditional indoor settings. Although not all studies directly compared indoor and outdoor contexts, the repeated emphasis on activity intensity underpins much of the rationale for conducting PE in outdoor settings [e.g., (52)]. However, this effect is often linked to the *type* of activity conducted (e.g., fitness classes outdoors leading to higher MVPA than classes indoors). These findings support the literature on the field that outdoor activity promotes higher intensity. Although it was not the scope of this paper, the patterns align with the health and behavioral measurement paradigm of PE, in which PE is a tool for promoting physical activity and to reduce sedentary behavior during the school day (11, 53).

Another trend of the data is the psychological and social benefits for using the outdoors in PE, including motivation, enjoyment, self-efficacy and stress. These outcomes were particularly prominent in research employing adventure-based, nature-based, or student-centered pedagogical approaches. Activities in outdoor swimming often show better settings for coping stress and safety awareness (21). The results also demonstrate that outdoors promote social interactions and social skills. These results include reducing disruptive behavior (37) or improved sense of community and relational skills (54). Although these findings point to potential affordances of outdoor environments for affective and motivational learning, heterogeneity in both constructs and measurement tools constrains direct comparison between studies. The pattern aligns with previous findings on green exercise, where staying in nature is connected to stress reduction and improving wellbeing (16).

An emerging strand of the literature frames outdoor PE as a means of supporting environmental education and sustainability (9). Research in this area has focused on activities such as plogging and environmentally themed outdoor learning units, examining outcomes related to environmental awareness, attitudes, and ecological competence (cognitive, emotional-value, and behavioral aspects) (55). Although these studies resonate with contemporary educational priorities, the evidence base remains narrow and methodologically diverse, suggesting a need for further conceptual refinement and greater consistency in research design. This highlights a growing purpose beyond traditional fitness or motor skill acquisition.

### Outdoor environments and activity types

This scoping review revealed considerably variations in the types of outdoor environments used for PE interventions. Natural and green environments, such as parks, forests, and beaches, were commonly used for activities focusing on fitness and navigation-based tasks. Blue environments, including open water and nautical centers, were used for water-based activities that addressed a combination of physical, psychological, social, and safety-related outcomes. In addition, many studies utilized built or semi-built outdoor spaces, such as schoolyards, sports fields, cycling paths, and nearby municipal facilities, highlighting the pragmatic use of school-adjacent environments.

The diversity of environments underscores the multifaceted nature of outdoor PE. Outdoor should therefore not just be understood as a homogenous context. The outdoor appear to function as flexible pedagogical spaces that are shaped by the curricular aims, available resources and the local conditions.

### Implications for research and practice

Before drawing on the findings presented here, practitioners and policymakers should note that this scoping review did not assess the methodological quality of included studies. As is standard in scoping review methodology, studies of varying design quality are presented side-by-side in the mapping, and the findings should therefore be interpreted with caution rather than as consolidated evidence base for practice. This scoping review indicates that outdoor PE is predominantly investigated as a strategy for increasing physical activity and fitness. While this aligns with public health priorities, it also reveals a narrow research focus. Future studies should extend beyond physical activity outcomes to further examine different pedagogical processes, learning mechanisms, and broader educational aims, including social and environmental learning. The review also displays an underrepresentation of older adolescents. Given the decline in physical activity during adolescence, this is an interesting gap for future research. More research is needed in upper secondary contexts, including longitudinal and mixed-methods designs that explore motivation, autonomy, and sustained participation. Greater conceptual clarity and detailed reporting of outdoor PE interventions would also strengthen comparability across studies. Beyond geographical expansion, future studies should consider engaging with locally grounded conceptual frameworks rather than simply transplanting Nordic or Anglo-American models into new settings. For instance, research in South American contexts could explore how existing cultural traditions relating nature, community, and physical activity, such as Indigenous land-based learning traditions, might inform alternative pedagogical approaches to outdoor PE that are meaningful within their own educational and cultural contexts (50). Such work would not only broaden the geographical reach of the field but enrich its conceptual foundations, moving outdoor PE research toward a genuinely cross-cultural evidence base.

For PE practice, the findings underscore the importance of access to nearby outdoor environments and the role of teacher autonomy in integrating outdoor lessons. For PE teachers, our findings suggest that relatively simple interventions, such as relocating a teaching unit to a local park or beach, has been shown to improve both physical fitness and gross motor coordination in primary school children (34), while incorporating plogging into secondary PE lessons has demonstrated improvements in students' environmental awareness and civic engagement (20). These examples suggest that outdoor PE does not require specialist facilities or extensive resources; rather, thoughtful use of accessible local environments, guided by clear pedagogical intentions, may be sufficient to extend the educational reach of PE beyond physical activity alone. Clear safety routines, especially in blue-space activities, and thoughtful sequencing of outdoor experiences across seasons may support coherent and sustainable implementation within school PE.

### Strengths and limitations

This scoping review has several strengths aligned with established methodological guidance for scoping reviews. This scoping review implemented a comprehensive and systematic search strategy across multiple databases spanning education, sport science, health, and psychology, supporting broad coverage of interdisciplinary research relevant to school-based PE. Additionally, using automatization tools as the ASreview together with human capabilities and independent screening by two reviewers strengthened the reliability of study selection and reduced selection bias (30). Several limitations should also be acknowledged. The review was restricted to peer-reviewed journal articles published in English, French, or Norwegian. Relevant studies published in other languages (Spanish, Portuguese, Mandarin, Arabic) or reported in grey literature (e.g., theses, reports, or practitioner evaluations) may therefore have been excluded, potentially contributing to the observed geographical concentration of the evidence base. This means that the Western dominance of this literature may partly reflect a linguistic sampling artefact rather than a genuine absence of outdoor PE practice in those regions. Another limitation is that no critical appraisal of methodological quality was conducted, which is typical for scoping reviews. Consequently, the findings should be understood as a descriptive mapping of the existing literature rather than an assessment of the strength or effectiveness of the evidence. In addition, pronounced heterogeneity in study designs, conceptualizations of outdoor PE, intervention characteristics, and outcome measures constrained comparability across studies and precluded any form of quantitative synthesis.

## Conclusions

This scoping review mapped forty-one empirical studies published between 2005 and 2025 investigating the use of outdoor environments in school-based PE. The data shows that outdoor PE is a growing but uneven field, with a discrepancy on research on older pupils and from non-Western contexts. Across studies, outdoor settings consistently supported higher levels of physical activity and improvements in several components of physical fitness, highlighting their value for promoting active engagement during PE. Psychological, social, and environmental outcomes, such as enjoyment, cooperation, motivation, and environmental awareness, were also reported. The review identified considerable diversity in the types of outdoor environments used, ranging from schoolyards and parks to forests, beaches, and blue-space locations. This diversity indicates that outdoor environments offer flexible pedagogical affordances, though these are not always purposefully aligned with curricular intentions. Overall, the findings suggest that outdoor PE holds substantial potential to support health, learning, and sustainability goals. Future research should address conceptual clarity, expand to underrepresented age groups and regions, and explore pedagogical mechanisms and long-term impacts to better inform practice and policy. To fully realize the potential of outdoor PE, however, this will require more than filling geographical and demographic gaps, it demands the development of clearer conceptual frameworks, deeper engagement with locally grounded educational traditions, and a genuine commitment to understanding the pedagogical mechanisms through which outdoor environments foster sustainable learning for health, wellbeing, and the planet.
