# Supplementary material for: The use of outdoor environments in school-based physical education: a scoping review
Source: Front Sports Act Living. 2026 Jun 23;8:1810598. doi: 10.3389/fspor.2026.1810598 (PMC13337431; doi:10.3389/fspor.2026.1810598)
Supplement: Supplementary file 1 [file Table1.docx]

**Table 1:** Summary Table of Included Studies

| Nr. | Author(s) (year) | Country | Participant characteristics | Research methods | Purpose of outdoor intervention | Outdoor-related variables | Activities | Outdoor environment type | Main findings |
| --- | --- | --- | --- | --- | --- | --- | --- | --- | --- |
| 1 | Bonavolontà et al., 2021 | Italy | 46 boys and girls (mean age 14.4 ± 0.3yrs; BMI 21.7 ± 0.5) | Interventional study (Quantitative) | Effects on body image perception and on body dissatisfaction in youngsters compared to peers | Body satisfaction and body-size perception | Walking and running | Fields near the school | Outdoor education has the potential to improve body image perception more effectively than traditional classes held in indoor facilities |
| 2 | Brusseau et al., 2016 | United States of America | 232 seventh and eighth grade students: 88 girls and 144 boys (mean age 13.3± 0.4 yrs) | A prospective and observational research design (Quantitative) | Effect of activity mode, environment, and semester on pedometer step counts/minute | Physical activity levels | Ball activities, ultimate frisbee, floor hockey, speedminton, capture the flag, aerobic. | A large grass field that was large enough for two full sized soccer fields. | OA displayed greater PA. 48,9% of activities of the year performed outdoors |
| 3 | Casado-Robles et al., 2022 | Spain | 179 students (52.5% females) from the 8.-9. grades (aged 13–15) | Cluster randomized controlled trial (Quantitative) | Compare the effects of two alternated teaching units (i.e., inside and outside the school center) and a traditional teaching unit | Knowledge test about the environment to practice PA; Perceived autonomy support. Self-determined motivation, PA and sedentary behavior. | Physical fitness and health tasks. Traditional and alternative games and sports (no more details) | Outside installations and features, green zones, or a municipal sport center | Innovative program improved students’ knowledge of the environment, improved perceived autonomy, motivation, intention to be more PA. No effect on objectively measured habitual MVPA and SB levels |
| 4 | Casey et al., 2011 | England | 28 secondary school boys in two classes (aged 14-16) | Teaching experiment (Qualitative) | Examined how students learned about and constructed their games concepts and operations as they 5designed their own g6ames using wikis | Learning experiences through game design | Invasion games created | Netball courts | The outdoor environment was used to create and apply different games by the students |
| 5 | Casey & Hastie, 2011 | England | 28 secondary school boys in two classes (aged 14-16) | Teaching experiment (Qualitative) | Ex7amined how stud8ents learned about9 making their own games and investigate their responses to the games-making process. | Learning experiences through game design | Invasion games created | Netball courts | Designing and playing their own outdoor games was recognized as a valid form of expression in physical education, even among disengaged students |
| 6 | Delextrat et al., 2020 | England | 307 pupils grade 8 (12–13 years old, 201 boys and 106 girls) | Cross-sectional design (Quantitative) | Investigate relationship between factors as gender, activity type, class and the amount of VPA and MVPA | Physical activity levels | ball games (basketball, football, handball, netball and rugby) and fitness activities | Not specified | Outdoor classes led to greater proportion of MVPA (Moderate to vigorous PA) and VPA (Vigorous PA) compared to indoor classes, and less time spent in sedentary PA. |
| 7 | Derigny et al., 2025 | France | 28 children (9.86 ± 0.65 years old; 15 girls and 13 boys) | Interventional study (Quantitative) | Assess an outdoor and cycling school-based intervention on the overall level of MVPA and on MVPA opportunities. | Physical activity levels | Cycling and other outdoor activities (no more details about them) | Park near the school and natural environments including forests and fields near the school | Overall MVPA (during the all day) was not impacted. Increase of MVPA during the PE lessons and during transport |
| 8 | Galotta et al., 2022 | Italy | 106 primary school children aged 7–11 years (grade 2, 3 and 5) | Interventional study (Quantitative) | Verify the feasibility and evaluate the efficacy of a PE intervention conducted in a natural environment | Height, weight, BMI, abdominal circumference, body fat, resting heart rate and BP, aerobic fitness, gross motor skills, PA level. | Aerobic activities such as walking, running, jumping, walking on their hands and knees, rolling, climbing | On the beach | PE intervention conducted in natural environment improve children’s physical fitness and gross motor coordination. The intervention also led to an increase of BMI |
| 9 | Gruno et Gibbons, 2024 | Canada | 2 PHE teachers and 34 students (no age or grade mentioned) | Interventional study with participatory action research (Qualitative) | Help students in PHE, make a connection between nature-based physical activity (NBPA) in PHE and being active in nature outside of school. | students’ experiences with, and perceptions of, NBPA both within their PHE lessons and outside of school | Walking, yoga, Gardening, Forest game, survival skills, geocaching, hiking, "Capture the flag", orienteering | The local mountain, lake and beach. Forest school campus, park adjacent to school grounds | Nature-based physical activity a vehicle for connecting with place. It led to interacting with local environment, engaging the senses. Provided space and the voice, to be willing and able to share insights about how they want to be active outdoors. |
| 10 | Guijarro-Romero et al., 2022 | Spain | 92 students (48% males and 52% females) in grade 7 and 8 (12-14 years). | Cluster-randomized controlled trial (Quantitative study) | Examine the effects of a PE-based reinforced program, through outdoor PA and body expression on cardiorespiratory fitness. Perceived and objective PA during a PE-based program | Cardiorespiratory fitness and  PA levels | Different track games, zumba choreographies | Not specified | A reinforced program, including outdoor PA and body expression, maintained level of cardiorespiratory fitness while making it possible to develop content other than physical fitness alone. |
| 11 | Jourand et al., 2018 | France | 16 students from grade 7 (about 12 years) | Qualitative case study (Qualitative) | Investigate interactions between the students and to point out their sequences in the dynamic according to different contexts in orienteering lesson. | Students’ experiences of cooperation | Orienteering | Forest | Three main modes of peer cooperation were found during orienteering lessons: co-construction, confrontation, and delegation. Outdoor PE contexts support dynamic social interactions and role distribution |
| 12 | Kwon et al., 2020 | United States of America | 2063 PE classes (Elementary, middle and high schools) | Observational study (SOFIT) (Quantitative) | Examine the association of PE-class characteristics, such as lesson context | PA levels | Not specified | Not specified | An outdoor PE lesson was significantly more likely than an indoor lesson to meet the recommended level of MVPA in elementary and middle schools, but not in high schools. |
| 13 | Lamoneda et al., 2024 | Spain | 170 students (Mean age = 16.06 ± 0.73 years; 43% boys, 57% girls) enrolled in year-11 (three groups) and year 12 (four groups) | A quasi-experimental, pre-test, post-test, comparison group research design (Mixed method) | Compare the effects of the hybridization of Outdoor Adventure Education (OAE) and Cooperative Learning vs Direct Instructions on students’ motivation and disruptive behavior. | Quantitative: Intrinsic Motivation in PE, disruptive behaviors Qualitative: students' and teachers' experiences | Orienteering | Parks, green areas, school’ sports center | Combined OAE and CL approaches had significantly higher intrinsic motivation, and significantly lower disruptive behaviors. CL-OAE hybrid orienteering learning unit identified 8 themes - most positive: enjoyment, autonomy, novelty and motivation; one negative: workload, three mixed themes: uncertainty, relationship and learning. |
| 14 | Lirgg et al., 2018 | United States of America | 41 grade 7 students (19 male and 22 female) (Mean = 12,68 years) | Interventional study (Quantitative) | Investigate the effect of a 3 week in-school bicycle program on the static balance, explosive leg power and agility | Static balance, explosive leg power, and agility | Cycling | Around the campus or in the adjoining neighborhoods on a primarily flat terrain | Bicycle riding may be an effective way to improve static balance ability, explosive leg power, and agility in children. |
| 15 | Lundhaug et al., 2025 | Norway | 33 children (13 girls and 20 boys), 8-12 years. | Qualitative case study (Qualitative) | Investigate how children experience stress, coping, and learning during outdoor swimming and water safety lessons. | Students’ experiences about stress, learning and coping | Swimming and water safety | A public beach (in the water) | Most children enjoyed the SWS lessons. They experienced stress differently. The teacher peers influenced the positive response outcome expectancies. Children developed knowledge and skills related to outdoor  SWS through collaborative learning. |
| 16 | Mandrillon et al., 2024 | France | 15 grade 6 students (7 boys, 8 girls) (Mean age boys 11,3 year and girls 11,1 years) | Interventional study (Mixed method) | Analyze student activity in traditional vs environmental orienteering. | Quantitative: PA levels (accelerometer) Qualitative: Students' experiences | Orienteering | Forest (5 lessons out of 6) and schoolyard (1 lesson out of 6) | Integrating environmental content into PE orienteering enhanced students’ PA and environmental awareness. |
| 17 | Martinez et al., 2025 | Spain | 143 students between 14 and 18 years (86 female; 53 male; 4 no gender; Mean age =15.58) | Interventional study (Quantitative) | Analyze the effect of a plogging didactic unit on the environmental awareness of the students in PE | Environmental awareness of the students | Plogging | Around the school | The experimental group improved their values in all the factors analyzed (motivation, social participation, media, education and environment) |
| 18 | Martinez et al., 2023 | Spain | 28 (16 females  and 12 males; 77.78% of the participants were 13 years old and 22.22% were 14 years). | Interventional study (Qualitative) | Integrate a plogging intervention within PE lessons to assess student perceptions of the activity | Students’ perspectives on the use of plogging in the educational context. | Plogging | No details for the first practical session; at the park Only for the second and third practical session. | Plogging heighten the environmental consciousness in secondary education, also after only 4 sessions |
| 19 | McKenzie et al., 2006 | United States of America | 36 public middle schools, an average enrollment of 1027 (SD = 285) students | Observational study  (Quantitative) | Assess girls’ (PA) in middle school PE as it relates to field site, lesson context and location, teacher gender, and class composition | PA levels, lesson context. | Not specified | Not specified | Outdoor lessons were 47 s shorter than indoor lessons but provided higher MVPA with greater intensity. Outdoor classes provided proportionally more time for fitness and management, whereas indoor lessons allocated more time for knowledge. |
| 20 | Mischenko et al., 2023 | Russia | 44 second grade students (7-9 years) | Interventional study (Quantitative) | Test a pedagogical mixed technology for developing environmental competence using PE methods and activities | Environmental competence, attitudes and behavior. Assessing how such integration influences  motor qualities | Quizzes with motor activity, relay races, outdoor and sports games | Forest park line | Cognitive, emotional-cognitive and behavioral  criteria of environmental competence significantly increased in EG children as well as their motor qualities |
| 21 | Molina-Garcia et al., 2016 | Spain | 189 students (60.8% girls; 16.3 years, SD ¼ 0.7) from 9 high schools | Observational study (Quantitative) | Analyze personal, psychosocial and environmental factors associated with moderate to vigorous PA (MVPA) during PE lessons. | Body height, weight and BMI PA levels, lesson context, physical self-efficacy perception | Not precisely described but activities mentioned: physical fitness activities, body expressions activities, games and sports | Not specified | The highest levels of MVPA were found when lessons took place outdoors |
| 22 | Pagels et al., 2016 | Sweden | 196 pupils (drawn from 4 schools), 2. graders (N=78, integrated 7-10 years), 5. graders (N=89, 11-13 years), 8. graders (N=29, 14-15 years) | Observational study (Quantitative) | Investigate differences between indoor and outdoor school time, and scheduled PE vs. free-living PA. PA during school time upon pupils’ PA during fall, late winter, and late spring | Body height, weight and BMI, PA levels, lesson context | Not specified | Not specified | PE outdoors generated higher physical activity levels, on any of the age levels |
| 23 | Pasek, 2021 | Poland | 220 students, EG=49 boys and 54 girls (Mean=11.26 years old)  CG=63 boys, 54 girls (11,28) | Interventional study (Quantitative) | Assess the impact of outdoor and indoor PE lessons on environmental attitudes over 2 years | Environmental attitudes | Not specified | Not specified | More outdoor lessons in PE led to an increase of environmental attitudes |
| 24 | Pasek et al., 2022 | Poland | 220 students, EG=49 boys and 54 girls (Mean=11.26(±0.32) years old)  CG=63 boys, 54 girls (11,28) | Interventional study (Quantitative) | Assess the impact of outdoor and indoor PE lessons on ecological knowledge over 2 years | Ecological knowledge | Not specified | Not specified | More outdoor lessons in PE led to an increase of ecological knowledge |
| 25 | Pasek et al., 2020 | Poland | 220 students, EG=49 boys and 54 girls (Mean=11.26(±0.32) years old)  CG=63 boys, 54 girls (11,28) | Interventional study (Quantitative) | Would an increase in the number of outdoor PE lessons increase the physical fitness. | International Physical Fitness Test with its individual components | Not specified | School facilities in close vicinity of school (no more details) | Students with regular outdoor PE lessons achieved significantly greater gains in running speed, leg power, and aerobic endurance, demonstrating outdoor activity’s stronger impact on overall physical fitness. |
| 26 | Pasek et al., 2014 | Poland | 220 students, EG=49 boys and 54 girls (Mean=11.26(±0.32) years old)  CG=63 boys, 54 girls (11,28) | Interventional study (Quantitative) | Investigate the influence of an increased number of outdoor lessons of PE on aerobic fitness and to evaluate students’ attitudes towards endurance efforts. | Mood before accomplishment of an effort test, endurance and subjective fatigue feeling. | Not specified | In the closest vicinity of school (no more details) | Students who had more outdoor PE lessons significantly improved endurance, felt less effort‐fatigue, and had better mood compared to indoor PE peers. |
| 27 | Polevoy, 2024 | Russia | 14 children (8-9 years) | Interventional study (Quantitative) | To develop a methodology for improving devolopment of coordination abilities in mentally retarded children aged 8-9 years in the lessons of adaptive physical education. | Coordination abilities | Outdoors games with the aim of developing motor abilities | Not specified | The use of outdoor games improved the coordination abilities |
| 28 | Polevoy et al., 2024 | Russia | 58 schoolchildren from 1st grade (7-8 years) | Interventional study (Quantitative) | Study the influence of outdoor games on the indicators of physical fitness | Physical fitness | Many outdoor games | Not specified | The use of outdoor games improved the physical fitness of children |
| 29 | Polevoy et al., 2024 (2) | Russia | 50 3rd grade students (9-10 years) | Interventional study (Quantitative) | To increase the level of endurance development in school children with the use of outdoor games | Endurance | Many outdoor games | Not specified | The use of outdoor games improved the endurance indicators. |
| 30 | Polevoy et al., 2023 | Russia | 56 schoolchildren from 2nd grade (8-9 years) | Interventional study (Quantitative) | Evaluate the impact of outdoor games on physical fitness indicators | Physical fitness | Many outdoor games | Not specified | The use of outdoor games improved the physical fitness of children in all tests |
| 31 | Polevoy et al., 2024 (3) | Russia | 133 1st graders (7-8 years) | Interventional study (Quantitative) | Assess the impact of the outdoor game named “Catch-up” on the outcomes of coordination abilities... | Coordination abilities; throwing, running, agility | Outdoor game named "Catch-up" | Not specified | The use of outdoor games improved the coordination abilities |
| 32 | Rocher et al., 2020 | Portugal | 595 students, 263 males, 330 females, 2 genderneutral (M-age= 14.02 years) | Case study (Mixed method) | Address the gap on nautical outdoor sports’ impact in PE regarding health, education, community, citizenship, social behavior, and environmental awareness. | Explore outdoor sports’ social benefits; well-being, education, citizenship, behavior, environment, and recall students’ feelings, thoughts, and experiences. | Surfing, canoeing, rowing, and sailing | The municipality’s nautical center. | Regular nautical outdoor sports in blue spaces significantly improve schoolchildren’s physical, mental, educational, social, and environmental well-being |
| 33 | Schlegel et al., 2022 | Czech Republic | 48 children (25 boys, 23 girls), 10–11 years | Interventional study (Quantitative) | To examine whether a four-week school-based Street Workout program improved children’s strength and flexibility | Flexibility  Pull and push strength, trunk strength and lower body strength | Street workout | Not specified | Street Workout proved to be an effective tool to develop strength, especially upper body. |
| 34 | Silva et al., 2022 | Portugal | 190 students: 108 boys (11,16 years), 80 girls (11.05 years), 2 gender neutral | Case study (Quantitative) | Explore the importance of surfing as a unique PE learning environment for children and adolescents. | Expectations, Self Confidence and Socialization in Surfing | Surfing | Surf center | Surfing PE improved self-confidence, maintained high individual expectations and socialization; over 90 % felt safe, had fun, and felt being part of a group. |
| 35 | Simonton et al., 2024 | United States of America | 72 9th grade students, 58% female; 42% male (Mage = 14.09) | Case study (Quantitative) | The study aimed to investigate how students’ perceived control, value, emotions, and future intentions vary across different PE content units | Perceived Control Beliefs Value Beliefs Student emotions Content-Specific Intention | Outdoor adventure challenges (hiking, geocaching, map reading, and team building) | Not specified | Students reported the adventure education unit as high. Students’ emotional connection to the content appears to be vital to their motivation, engagement, and intention to engage in the activity outside of PE |
| 36 | Skala et al., 2012 | United States of America | 211 3rd, 4th and 5th-graders from 74 schools representing 6740 students | Observational study (SOFIT) (Quantitative) | Examine the associations between specific environmental characteristics (teacher, classroom, and lesson context) and MVPA in elementary school children during PE class. | Time and percentage spent on lesson context, time and percentage engaged in MVPA | Not specified | Not specified | Outdoor lessons significantly increased MVPA, while indoor classes were more sedentary, emphasizing knowledge and skills. Indoors involved sitting; outdoors prioritized active play and game participation. |
| 37 | Somboonwong et al., 2012 | Thailand | 457 schoolboys from grades 1 to 6, (5,5-12 years) | Observational study (Quantitative) | Determine thermoregulatory, cardiovascular responses and heat illness in children exercising outdoors in PE class under hot and humid climate. | Exercise duration and intensity Thermoregulatory and cardiovascular responses Hydration status, occurrence of heat related illness, body weight, height and BMI | Different ball games for each grade and athletics, e.g. chair ball, soccer, mini-rugby, handball | Not specified | Students maintained cardiovascular function outdoors, but primary schoolchildren, especially overweight and dehydrated, faced increased risk of heat illness. |
| 38 | Stuhr et al., 2015 | United States of America | 94 7th-8th grade students | Case study (Qualitative) | Investigate middle school students’ perceptions of intrapersonal and interpersonal relationship skills (IIRS) during a 15-lesson adventure-based learning (ABL) unit in physical education | 5 IIRS: active listening, helping others, respect for others, encouraging others and leadership | Several outdoor games linked to the IIRS | Not specified | The study found students connected with, valued, developed, and transferred relationship skills from adventure-based learning to PE, sports, and school contexts |
| 39 | Yfantidou et al., 2025 | Greece | 74 students, 5th grade of primary school, 39 girls, 35 boys (10.27 ± 0.23 years) | Interventional study (Quantitative) | Assess the effectiveness of an intervention program to cultivate environmental awareness | Environmental knowledge | Walk, Environmental games, Treasure hunt game, Problem-solving games, role-playing | Schoolyard, a nearby park and a forest | PE interventions a meaning ful strategy to promote sustainability, fostering young tourists as responsible consumers respecting and protecting nature. |
| 40 | Zhang et al., 2020 | China | 51 7th grade students, 24 boys and 27 girls (12 years) | Interventional study (Quantitative) | Evaluate the effect of an 8-week theory-based intervention on adolescents´ psychological determinants and physical activity participation. | Exercise attitude, subjective norms, perceived behavioral control, and exercise intention Self efficacy and outcome expectancy. PA level | Basketball | Playground | The combined theory-based intervention significantly improved adolescents’ attitudes, self-efficacy, intentions, and objectively measured physical activity compared with the control group |
| 41 | Zhou et al., 2025 | China | 4482 students (Grades 1 to 9) | Observational study (SOFIT)  (Quantitative) | Explore the association between class-level factors, such as lesson start time, class size, lesson location, PE content, and PE context, and student engagement in MVPA during PE lessons in both elementary and middle schools. | PA levels; lesson context | Not specified | School fields and basketball courts | Outdoor PE lessons showed higher MVPA percentages compared to indoor lessons in middle school |

*Note*: References of the included papers can be found in the Appendix 2
