# Supplementary material for: The use of outdoor environments in school-based physical education: a scoping review
Source: Front Sports Act Living. 2026 Jun 23;8:1810598. doi: 10.3389/fspor.2026.1810598 (PMC13337431; doi:10.3389/fspor.2026.1810598)
Supplement: Supplementary file 2 [file Supplementaryfile1.docx]

Supplementary Material

# Appendix 1: Supplementary Data - The full search string

Scopus (database: Scopus). Search conducted on June 17, 2025: ( "outdoor" OR "outdoor education" OR "outdoor learning" OR "outdoor activities" OR "outdoor teaching" OR "environmental education" OR "outside the classroom" OR "out-of-classroom" OR "excursion" OR "field

trip" OR "fieldtrip" OR "fieldwork" OR "field work" OR "place-based education" OR "place-based learning" OR "place-based teaching" OR "green exercise" OR "blue exercise" ) AND ( "physical education" OR "PE" ) AND PUBYEAR > 2004 AND PUBYEAR < 2026

EBSCO (databases: Education Source, ERIC, MEDLINE, Psychology and Behavioral Sciences Collection and SPORTDiscus). Search conducted on June 17, 2025: (outdoor OR outdoor education OR outdoor learning OR outdoor activities OR outdoor teaching OR environmental education OR outside the classroom OR out-of-classroom OR excursion OR field trip OR fieldtrip OR fieldwork OR field work OR place-based education OR place-based learning OR place-based teaching OR green exercise OR blue exercise) AND (physical education OR PE) AND (PV 2005-2025)
