# Supplementary material for: The use of outdoor environments in school-based physical education: a scoping review
Source: Front Sports Act Living. 2026 Jun 23;8:1810598. doi: 10.3389/fspor.2026.1810598 (PMC13337431; doi:10.3389/fspor.2026.1810598)
Supplement: Supplementary file 3 [file Supplementaryfile2.docx]

Supplementary Material

# Appendix 2. Supplementary Data. The included studies in the scoping review

1. Bonavolontà V, Cataldi S, Fischetti F. Changes in body image perception after an outdoor physical education program. J Phys Educ Sport. (2021) 21:632-637. doi: 10.7752/jpes.2021.s1074

2. Brusseau TA, Burns RD, Fu Y. Contextual factors related to physical activity during daily middle school physical education. J Sci Med Sport. (2016) 19:733-737. doi: 10.1016/j.jsams.2015.10.001

3. Casado-Robles C, Viciana J, Guijarro-Romero S, Mayorga-Vega D. Effect of an inside-outside school alternated teaching unit of knowledge of the environment for practicing physical activity: a cluster randomized control trial. J Teach Phys Educ. (2022) 41:149-158. doi: 10.1123/jtpe.2020-0132

4. Casey A, Hastie P, Rovegno I. Student learning during a unit of student-designed games. Phys Educ Sport Pedagogy. (2011) 16:331-350. doi: 10.1080/17408989.2011.557654

5. Casey A, Hastie P. Students and teacher responses to a unit of student-designed games. Phys Educ Sport Pedagogy. (2011) 16:295-312. doi: 10.1080/17408989.2010.535253

6. Delextrat A, Esser P, Beale N, Bozon F, Eldridge E, Izadi H, et al. Effects of gender, activity type, class location and class composition on physical activity levels experienced during physical education classes in British secondary schools: a pilot cross-sectional study. BMC Public Health. (2020) 20:1590. doi: 10.1186/s12889-020-09698-y

7. Derigny T, Khan MAU, Mura M, Hamadene T, Bouyat M, Valerio G, et al. The “Écolo’coteaux” programme: an outdoor and cycling school-based intervention on daily physical activities opportunities. Phys Educ Sport Pedagogy. (2025). doi: 10.1080/17408989.2025.2468929

8. Gallotta MC, Zimatore G, Cardinali L, Falcioni L, Bonavolontà V, Curzi D, et al. Physical education on the beach: an alternative way to improve primary school children's skill- and health-related outcomes during the COVID-19 pandemic. Int J Environ Res Public Health. (2022) 19:3680. doi: 10.3390/ijerph19063680

9. Gruno J, Gibbons S. Using their (photo)voice: student experiences with nature-based physical activities in and beyond physical and health education. Can J Action Res. (2024) 24:71-104. doi: 10.33524/cjar.v24i3.674

10. Guijarro-Romero S, Mayorga-Vega D, Casado-Robles C, Viciana J. School physical education-based reinforced program through moderate-to-vigorous physical activity improves and maintains schoolchildren's cardiorespiratory fitness: a cluster-randomized controlled trial. Sci Sports. (2022) 37:74.e1-74.e12. doi: 10.1016/j.scispo.2020.12.011

11. Jourand C, Adé D, Sève C, Komar J, Thouvarecq R. Dynamics of student interactions: an empirical study of orienteering lessons in physical education. Phys Educ Sport Pedagogy. (2018) 23:134-149. doi: 10.1080/17408989.2017.1342790

12. Kwon S, Welch S, Mason M. Physical education environment and student physical activity levels in low-income communities. BMC Public Health. (2020) 20:147. doi: 10.1186/s12889-020-8278-8

13. Lamoneda J, González-Víllora S, Evangelio C, Fernandez-Rio J. Hybridizing outdoor adventure education and cooperative learning in physical education: students and teachers' views. J Adventure Educ Outdoor Learn. (2024) 24:159-174. doi: 10.1080/14729679.2022.2087194

14. Lirgg CD, Gorman DR, Merrie MD, Hadadi AA. Effect of a bicycling unit on the fitness of middle school students. Phys Educ. (2018) 75:165-174. doi: 10.18666/TPE-2018-V75-I2-7786

15. Lundhaug T, Moe VF, Eriksen HR. Children’s experiences of stress, coping, and learning during outdoor swimming and water safety lessons. J Experiential Educ. (2025). doi: 10.1177/10538259251325175

16. Mandrillon K, Gottsmann L, Desplanques F. Towards an integration of physical activity and environmental awareness: analysis of students’ activity in physical education. Phys Educ Sport Pedagogy. (2024). doi: 10.1080/17408989.2024.2319059

17. Martinez-Mirambell C, García-Taibo O, Ferriz-Valero A, Baena-Morales S. Plogging improves environmental awareness in high school physical education students. J Adventure Educ Outdoor Learn. (2025) 25:333-343. doi: 10.1080/14729679.2023.2235705

18. Martínez-Mirambell C, Boned-Gómez S, Urrea-Solano M, Baena-Morales S. Step by step towards a greener future: the role of plogging in educating tomorrow’s citizens. Sustainability. (2023) 15:13558. doi: 10.3390/su151813558

19. McKenzie TL, Catellier DJ, Conway T. Girls' activity levels and lesson contexts in middle school PE: TAAG baseline. Med Sci Sports Exerc. (2006) 38:1229-1235. doi: 10.1249/01.mss.0000227307.34149.f3

20. Mischenko NY, Kolokoltsev M, Romanova E, Bayankin O, Kispayev T, Vrachinskaya T, et al. Mixt-technology for the development of environmental competence in physical education classes in 7-9-years-old children. J Phys Educ Sport. (2023) 23:52-58. doi: 10.7752/jpes.2023.01006

21. Molina-García J, Queralt A, Estevan I, Sallis JF. Ecological correlates of Spanish adolescents’ physical activity during physical education classes. Eur Phys Educ Rev. (2016) 22:479-489. doi: 10.1177/1356336X15623494

22. Pagels P, Raustorp A, Guban P, Fröberg A, Boldemann C. Compulsory school in- and outdoors—implications for school children's physical activity and health during one academic year. Int J Environ Res Public Health. (2016) 13:699. doi: 10.3390/ijerph13070699

23. Pasek M. Outdoor vs indoor physical education lessons as an opportunity to shape environmental attitudes. Balt J Health Phys Act. (2021) 13:43-51. doi: 10.29359/BJHPA.13.Spec.Iss1.04

24. Pasek M, Bendíková E, Kuska M, Żukowska H, Dróżdż R, Olszewski-Strzyżowski DJ, et al. Environmental knowledge of participants’ outdoor and indoor physical education lessons as an example of implementing sustainable development strategies. Sustainability. (2022) 14:544. doi: 10.3390/su14010544

25. Pasek M, Szark-Eckardt M, Wilk B, Zuzda J, Żukowska H, Opanowska M, et al. Physical fitness as part of the health and well-being of students participating in physical education lessons indoors and outdoors. Int J Environ Res Public Health. (2020) 17:309. doi: 10.3390/ijerph17010309

26. Pasek M, Michalowska-Sawczyn M, Nowak-Zaleska A. Changes in maximal aerobic fitness and students' attitude towards physical effort during outdoor and indoor school lessons of physical education. Balt J Health Phys Act. (2014) 6:24-33. doi: 10.2478/bjha-2014-0003

27. Polevoy G. Development of coordination abilities of mentally retarded children in physical education lessons. Bangladesh J Med Sci. (2024) 23:1219-1226. doi: 10.3329/bjms.v23i4.76552

28. Polevoy G, Sablin AB, Fuentes-Barría H, Aguilera-Eguía E. The influence of outdoor games on the development of physical qualities in children 7-8 years old in physical education lessons. Ann Appl Sport Sci. (2024) 12:e1260. doi: 10.61186/aassjournal.1260

29. Polevoy GG, Sablin AB, Chernishev SV. Development of endurance in physical education lessons with the use of outdoor games. Bangladesh J Med Sci. (2024) 23:254-261. doi: 10.3329/bjms.v23i1.70758

30. Polevoy GG, Egorycheva EV, Fedorov AV, Ardigò LP. Outdoor games – an effective means of developing physical qualities of junior schoolchildren. Theory Pract Phys Cult. (2023) (12):55-57.

31. Polevoy G, Ouergui I, Padulo J, Ardigò LP. The outdoor game “catch-up” is a tool to support the development of coordination abilities in children. Ann Appl Sport Sci. (2024) 12:e1264. doi: 10.61186/aassjournal.1264

32. Rocher M, Silva B, Cruz G, Bentes R, Lloret J, Inglés E. Benefits of outdoor sports in blue spaces: the case of school nautical activities in Viana do Castelo. Int J Environ Res Public Health. (2020) 17:8470. doi: 10.3390/ijerph17228470

33. Schlegel P, Sedláková L, Křehký A. Street workout is the new gymnastics — strength development in a very short school-based program. J Phys Educ Sport. (2022) 22:489-494. doi: 10.7752/jpes.2022.02061

34. Silva B, Cruz G, Bentes R, Lima RF. Surfing on physical education curriculum and the impact on student’s well-being. J Phys Educ (Maringa). (2022) 33:e3317. doi: 10.4025/jphyseduc.v33i1.3317

35. Simonton KL, Garn AC, Layne TE. An investigation of content-specific unit emotions in secondary physical education. Educ Sci. (2024) 14:1123. doi: 10.3390/educsci14101123

36. Skala KA, Springer AE, Sharma SV, Hoelscher DM, Kelder SH. Environmental characteristics and student physical activity in PE class: findings from two large urban areas of Texas. J Phys Act Health. (2012) 9:481-491. doi: 10.1123/jpah.9.4.481

37. Somboonwong J, Sanguanrungsirikul S, Pitayanon C. Heat illness surveillance in schoolboys participating in physical education class in tropical climate: an analytical prospective descriptive study. BMJ Open. (2012) 2:e000741. doi: 10.1136/bmjopen-2011-000741

38. Stuhr PT, Sutherland S, Ressler J, Ortiz-Stuhr EM. Students' perception of relationship skills during an adventure-based learning unit within physical education. J Adventure Educ Outdoor Learn. (2015) 18:27-38.

39. Yfantidou G, Kouli O, Morela E, Kouli E. Enhancing environmental awareness in tourism through movement: a physical education approach. Educ Sci. (2025) 15:297. doi: 10.3390/educsci15030297

40. Zhang Y, Yin Y, Liu J, Yang M, Liu Z, Ma X. Impact of combined theory-based intervention on psychological effects and physical activity among Chinese adolescents. Int J Environ Res Public Health. (2020) 17:3026. doi: 10.3390/ijerph17093026

41. Zhou Y, Wang L, Chen R, Wang B. Associations between class-level factors and student physical activity during physical education lessons in China. Int J Behav Nutr Phys Act. (2025) 22:1. doi: 10.1186/s12966-024-01703-6
